# Unravelling the Evolutionary Dynamics of High-Risk *Klebsiella pneumoniae* ST147 Clones: Insights from Comparative Pangenome Analysis

**DOI:** 10.3390/genes14051037

**Published:** 2023-05-02

**Authors:** Suchanda Dey, Mahendra Gaur, Ellen M. E. Sykes, Monica Prusty, Selvakumar Elangovan, Sangita Dixit, Sanghamitra Pati, Ayush Kumar, Enketeswara Subudhi

**Affiliations:** 1 Centre for Biotechnology, School of Pharmaceutical Sciences, Siksha ‘O’ Anusandhan (Deemed to be University), Bhubaneswar 751003, India; suchandadey1993@gmail.com (S.D.);; 2 Department of Microbiology, University of Manitoba, Winnipeg, MB R3T2N2, Canada; 3 School of Biotechnology, Kalinga Institute of Industrial Technology, Bhubaneswar 751024, India; 4 Regional Medical Research Centre, Bhubaneswar 751023, India; drsanghamitra12@gmail.com

**Keywords:** *Klebsiella pneumoniae*, high-risk clone, ST147, pangenome analysis, SNP

## Abstract

Background: The high prevalence and rapid emergence of antibiotic resistance in high-risk *Klebsiella pneumoniae (KP)* ST147 clones is a global health concern and warrants molecular surveillance. Methods: A pangenome analysis was performed using publicly available ST147 complete genomes. The characteristics and evolutionary relationships among ST147 members were investigated through a Bayesian phylogenetic analysis. Results: The large number of accessory genes in the pangenome indicates genome plasticity and openness. Seventy-two antibiotic resistance genes were found to be linked with antibiotic inactivation, efflux, and target alteration. The exclusive detection of the *bla*_OXA-232_ gene within the ColKp3 plasmid of KP_SDL79 suggests its acquisition through horizontal gene transfer. The association of seventy-six virulence genes with the *acrAB* efflux pump, T6SS system and type I secretion system describes its pathogenicity. The presence of Tn*6170*, a putative Tn7-like transposon in KP_SDL79 with an insertion at the flanking region of the *tnsB* gene, establishes its transmission ability. The Bayesian phylogenetic analysis estimates ST147’s initial divergence in 1951 and the most recent common ancestor for the entire *KP* population in 1621. Conclusions: Present study highlights the genetic diversity and evolutionary dynamics of high-risk clones of *K. pneumoniae*. Further inter-clonal diversity studies will help us understand its outbreak more precisely and pave the way for therapeutic interventions.

## 1. Introduction

Antimicrobial resistance (AMR) is a serious global threat to human health, as the World Health Organization (WHO) has stated. In 2019, bacterial AMR was responsible for an estimated 541,000 deaths globally, with 133,000 occurring in the WHO European region alone. By 2050, the number of deaths attributed to bacterial AMR is projected to reach 10 million [1]. Multidrug-resistant (MDR) *Klebsiella pneumoniae* (*Kp*) is a critical public health issue and has been designated as a top-priority pathogen by the WHO [2]. *Kp*, a member of the *Enterobacteriaceae* family, is responsible for a growing number of hospital-acquired infections, leading to significant morbidity and mortality [3]. In the United States alone, *Klebsiella* species are responsible for nearly 600 deaths annually [4]. The upsurge in sequence type 147 (ST147) has driven the rapid emergence of MDR *Kp*, a high-risk (HiR) international clone. This was first identified in Hungary between 2008 and 2010 and has since spread geographically [5]. A literature survey on PubMed in 2021 using the terms “*Klebsiella pneumoniae*” and “ST147” revealed 171 globally relevant studies, with a significant surge from 2013 to 2021, displaying its increasing significance.

Nonetheless, the investigations on this clone have been mainly confined to case studies from hospitals in over 23 countries [5]. Limited reports on this clone’s global prevalence, pangenome analysis, and population dynamics pose challenges in comprehending its emergence. The mortality rate linked to this clone is relatively high, ranging from 48 to 59% globally [6]. Recently, the global emergence of ST147 has been documented, and its phylogenetic context of antimicrobial and virulence factors has been explored [7]. However, the major limitations of these other studies were that the genomes analyzed were partially assembled and existed at a ‘draft’ or ‘scaffold’ level. This can impede the identification of critical AMR determinants and hinder tracking their spread across bacterial strains. Therefore, including fully resolved genome sequences in the pangenome analysis is crucial, as this provides a more accurate representation of the genetic information and offers us an ultimate resolution to discriminate among highly related pathogens [8].

Despite several studies on this clone, a systematic analysis and mining of the genomic information for this clone, especially concerning its pangenome analysis, is lacking. Hence, a comprehensive pangenome analysis of fully resolved genome sequences is crucial to providing accurate genetic information and insights into its bacterial evolution, population structure, host interaction, and niche adaptation [9]. The focus of the previous study was on the genome-wide distribution of AMR genes and virulence factors in an extremely drug-resistant (XDR) strain belonging to ST147 (KP_SDL79) [10]. Through a pangenome analysis, this study aimed to investigate the genome plasticity and population diversity within ST147 populations to gain better insight into this clonal type. Additionally, we characterized the genomes by analyzing drug resistance and virulence factors, plasmid profiles, transposons, biosynthetic gene clusters (BGCs), and single-nucleotide polymorphisms (SNPs) using various bioinformatics tools. The study also aimed to provide insights into the evolution and global spread of these HiR clones. The findings have the potential to provide significant insights for controlling the spread of HiR *Kp* clones and effective strategies to manage their transmission.

## 2. Materials and Methods

### 2.1. Genome Reconstruction, Finishing and Quality Assessment of KP_SDL79

The raw reads obtained from the previous whole genome sequencing study [10] were checked for quality and trimmed using FastQC v0.11.8. [11] and Trimmomatic v0.36 (Phred score > 20) [12]. De novo assembly was performed using four different assembly tools: Spades v3.15.4 [13], Unicycler v0.5.0 [14], SOAPdenovo [15] and AbSys v2.1.5 [16]. A range of k-mer values was chosen according to the user manual of the individual assembly tools. The presence of conserved genes in the assembled contigs was evaluated using BUSCO v5.3.2 [17], and the quality was assessed on 25 January 2021, using QUAST 4.0 (https://github.com/ablab/quast, accessed on 25 January 2021) and CheckM v1.0.18 (https://github.com/Ecogenomics/CheckM, accessed on 25 January 2021). Repeat masking and scaffolding was performed using RepeatMasker v4.0.9 with default parameters [18] and Ragout v2.3 [19]. Based on its quality assessment, the best-quality assembly was derived from Unicycler and was subsequently used in downstream analysis. The discrepancies in Unicycler assembly were determined by aligning quality-filtered reads back to the contigs using Bowtie2 v12.3 with default parameters [20].

### 2.2. Acquisition of Genomic Sequences from Public Databases and Data Analysis

A total of 315 *Kp* genomes were retrieved from NCBI on 15 Febuary 2021, including 40 complete, 100 scaffold, and 175 draft assemblies. Filtering was based on complete genomes with contig numbers within 2.5 times the median and CDS and genome length within three standard deviations of the mean. The selection of genomes was based on recent studies, which have shown that high-quality genomes play a crucial role in pangenome and genome mining analyses [21]. Therefore, draft and scaffold level assemblies were excluded from this study. The capsular serotypes (K-type and O-type) were determined using the Kaptive tool v0.7.3 (https://github.com/klebgenomics/Kaptive, accessed on 25 January 2021).

### 2.3. Genome Similarity Estimation

The degree of genetic relatedness between the 41 genomes was determined using the MinHash algorithm. Mash [22] calculated “mash dist” with a k-mer size of 21 and sketches of size 5000. The output was converted into a distance matrix with assembly accession numbers as columns and rows. The Mash distance values were normalized between 0 to 1 (0 = identical sequence; 1 = dissimilar sequence). R packages ggplot2, ggh4x (https://github.com/teunbrand/ggh4x, accessed on 25 January 2021) and hclust were used to generate a clustered heat map based on average-linkage unweighted pair group method with arithmetic mean (UPGMA) using Euclidean distance method from the pairwise Mash matrix. The elbow method [23] using the K-means algorithm (nstart = 25, iter.max = 1000) was used to determine the optimal number of clusters by plotting the within-cluster sum of squared errors (WSS) versus a number of clusters.

### 2.4. Pangenome Analysis and Functional Annotation

Pangenome analysis was performed with the Roary pipeline [24]. All other Roary parameters were set to default. Minimum blastp identity was set to 90% and the inflation value for the Markov clustering technique was set at 1.5. A gene presence/absence-derived file from Roary analysis was used to visualize distributions of the pangenome in the isolates. The Roary2SVG script was used to plot pangenome distributions to individual isolates. A maximum-likelihood (ML) phylogenetic tree was generated on the core genome alignment using the Randomized Axelerated Maximum Likelihood method (RAxML) [25] and visualized using iTol v2.10 web server (https://itol.embl.de/, accessed on 25 January 2021). A hierarchical Bayesian clustering algorithm was implemented in Fastbaps v1.0 (Fast Hierarchical Bayesian Analysis of Population Structure) in R v3.5.3 with packages ape v5.3, ggplot2 v3.1.1, ggtree v2.4.1, maps v3.3.0 and phytools v6.60 to cluster the core genome SNPs as a sparse matrix [26]. The Clusters of Orthologous Genes (COG) and Kyoto Encyclopedia of Genes and Genomes (KEGG) databases were implemented in BPGA v1.3 for functional annotations of core, accessory and unique genes [27]. The orthologous genes of KP_SDL79 and their closely related isolates were determined using OrthoVenn2 at an e-value cut-off of 1 × 10^−5^ and MCL inflation of 1.5 [28].

### 2.5. SNP Analysis and Phylogeny Reconstruction

Core SNPs were identified using Snippy v4.6 by aligning the reads from each genome against the reference (GCA_016903735) (https://github.com/tseemann/snippy, accessed on 25 January 2021). SnpEff v5.1 [29] annotated high-quality SNPs and indels after deleting low-confidence alleles with a consensus base quality of <20 and a read depth of <5 or a heterozygous base call. The parsimony tree was constructed using consensus core genome sequences from both Snippy and kSNP3 v3.0 [30]. The tree was created as a consensus of up to 100 equally parsimonious trees using the SNP matrix file (SNPs all matrix.fasta) produced by the tool. The optimal k-mer size was set at 19 using the *K-chooser* available with the package. The tree was visualized in FigTree v1.4.4 (http://tree.bio.ed.ac.uk/software/figtree/, accessed on 25 January 2021).

### 2.6. BEAST Analysis

The recombination regions in the core genes were identified and filtered using Gubbins v2.4.1 [31]. Based on non-recombining core genome sequences, the divergence times of the isolates were estimated using Bayesian Evolutionary Analysis Sampling Trees (BEAST) v1.10.4. Root-to-tip distances and regression analysis were computed using TempEst v1.5.1. The best-fitting model priors were defined by testing the combination of two molecular clock models (strict and uncorrelated relaxed), demographic models (Bayesian Skyride and Bayesian Skyline plot) with a general time-reversible (GTR + γ) substitution model of evolution. Each demographic molecular clock combination was run for 100 million states to check the convergence in the datasets assessed using Tracer v1.7.1. Finally, a time tree was generated using TreeAnnotator from the best-fitting model having effective sample size (ESS) values of more than 200. The tree was visualized using FigTree.

### 2.7. Association of AMR, Virulence Determinants and Plasmids

To compare AMR genes, virulence and plasmid replicon, ABRicate (https://github.com/tseemann/abricate, accessed on 25 January 2021) local reference databases were used: Comprehensive Antibiotic Resistance Database (CARD) (https://card.mcmaster.ca/, accessed on 25 January 2021), VirulenceFinder [32], Virulence Finder Database (VFDB) (http://www.mgc.ac.cn/VFs/, accessed on 25 January 2021) and PlasmidFinder [33]. The plasmid Sankey plot was generated using SankeyMATIC software (https://sankeymatic.com/, accessed on 25 January 2021). The locally collinear blocks (LCBs) were determined by Mauve [31] and synteny plots were inferred by DNASTAR (https://www.dnastar.com/, accessed on 25 January 2021) to determine the significant difference between the plasmids.

### 2.8. Genome Mining and Identification of Essential Genomic Elements

AntiSMASH v6.0 [34] was used to predict biological gene clusters (BGCs) and ISsaga2 in ISFINDER and determine the insertion sequences (ISs) [35]. The detection strictness was eased in antiSMASH, and extra features, such as ClusterBlast, Cluster Pfam analysis, and Pfam-based Gene Ontology (GO) term annotation, were enabled. The CRISPRCasFinder webserver predicted CRISPR elements and spacers [36]. The CRISPR arrays from each genome were counted with an evidence level ≥ 1 and assigned to them. The Prophage Hunter web tool was used to forecast prophage elements [37].

### 2.9. Statistical Analysis

In conjunction with K-means clustering, elbow statistics were used to determine the optimal number of clusters for genetic relatedness among the strains, as described in Section 2.3. Markov chain Monte Carlo (MCMC) simulations were performed in triplicates for 100 million steps with sampling every 10 generations. To ensure the convergence of the simulations, a cut-off of 100 was set for the effective sample size (ESS) of crucial parameters, including the substitution rate, tree root height and population size (https://github.com/beast-dev/beast-mcmc, accessed on 25 January 2021).

## 3. Results

The characteristics and evolutionary relationships among the ST147 members were examined by analyzing 41 genome sequences using comparative genomics. The genomes were obtained from a public database (https://www.ncbi.nlm.nih.gov/, accessed on 25 January 2021), and one genome (KP_SDL79, accession: JAMXTJ000000000) from previous research was included for a further downstream analysis [10]. Summaries of the genome statistics and metadata are presented in Table 1.

### 3.1. Improvement of Draft Assembly of KP_SDL79

The sequencing run generated 10.3 million reads with an average Phred quality score of 37.8. Quality trimming reduced the reads to 7 million, assembled with an optimized k-mer length (Table 2). The contigs were rearranged into scaffolds and reordered based on the strains’ relatedness. The genome size was 5,622,734 bp distributed into 43 scaffolds (≥200 bp) with a genome coverage of 96.66%, an N50 value of 218,233 bp and a GC content of 57.21%.

### 3.2. Dataset Description and Genome Statistics

A total of 315 genome sequences were retrieved from a public repository. After we reduced the redundancy and conducted quality checks, 40 complete genomes were selected and re-annotated. The prokka annotation of these genomes revealed an overall genome size ranging from 5,344,576 to 6,109,775 bp, with an average GC content of 57.3741%. The number of coding sequences (CDS) per genome ranged from 4946 to 5828. The annotation also determined 31 rRNA genes, 103 tRNA genes and 1 tmRNA gene in each genome (Table 1).

### 3.3. Genomic Relatedness Analysis Using Mash Distance

**Figure 1:**
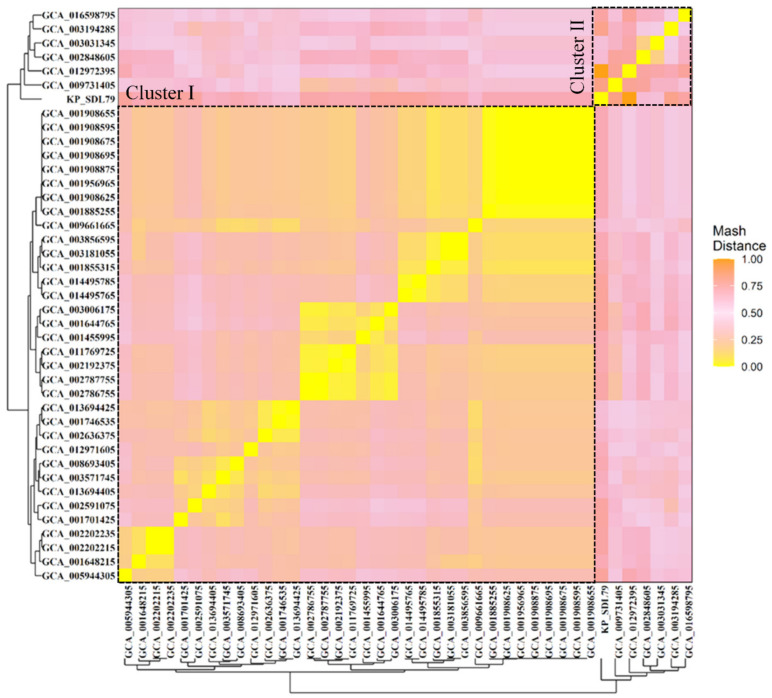
Clustered heatmap representation of genetic relatedness across the 41 genomes using Mash distance. The pairwise similarity between the samples is scaled from 0 (yellow) to 1 (orange), representing the highest and lowest genetic similarity between the genomes. The mash clustering identified 2 major clusters marked with a black dotted line. KP_SDL79 is found in cluster II along with six other isolates.

A hierarchical clustering analysis was performed on all 41 genomes using a distance matrix based on their genome similarity. The results showed that the average pairwise Mash distance between all strains was 0.0012 ± 0.0007, indicating genetic relatedness among the members of the same sequence type. KP_SDL79 and six other strains (GCA_002848605, GCA_003031345, GCA_003194285, GCA_009731405, GCA_012972395 and GCA_016598795) formed a distinct cluster, separated from the remaining 34 strains in cluster II (Figure 1). This ladder-like branching pattern suggests a descendant relationship among the strains.

### 3.4. Insights into Pangenome Structure, Core Phylogenetic Relationships and Functional Characterization

**Figure 2:**
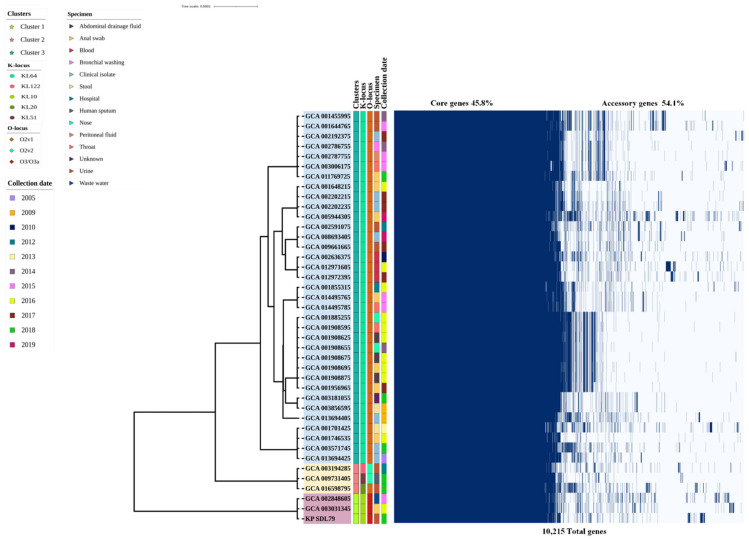
Core gene phylogenetic analysis and matrix visualization of 41 analyzed ST147 genomes. The pangenome presence/absence matrix visualization, metadata detailing each genome’s isolation source, clusters from Fastbaps (blue, yellow and pink), collection dates, K and O-locus of all the strains were created using Roary and iTOL.

The pangenome analysis revealed that the set of 10,215 genes comprised 4406 and 279 core genes (99% ≤ strains ≤ 100%) and soft-core genes (95% ≤ strains < 99%) followed by 1478 shell genes (15% ≤ strains < 95%) and 4052 cloud genes (0% ≤ strains < 15%), respectively. Only 45.8% belonged to the core genes, while 54.1% were accessory genes (Figure S1A). The core genome stabilized quickly with the first five genomes, but adding more genomes led to high genomic plasticity, indicating an “open” pangenome structure (Figure S1B) [38]. The phylogenetic analysis of the core genes showed that the isolates were divided into three clusters (clusters 1–3). The KP_SDL79 isolates from urine were grouped with strains from wastewater and anal swabs in cluster I, indicating its specificity to those environments, while most of the isolates (n = 35) were in cluster III, and only three were in cluster II (Figure 2). COG and KEGG categories were assigned to the pan genes with different functional classes and varying percentages, as depicted in Figure S2A,B. The COG categories revealed that the majority of the core genomes were associated with “R” (12.7%, general function prediction only) followed by “E” (11.4%, amino acid transport and metabolism), “G” (10.6%, carbohydrate transport and metabolism), “K” (9.4%, transcription) and “P” (7.75%, inorganic ion transport and metabolism), whereas the accessory and unique genes were associated with “L” (17.2%, replication, recombination and repair), “R” (13%), “S” (10%, function unknown) and “K” (9.8%), respectively. Similarly, the KEGG analysis revealed that the core genes were primarily related to carbohydrate metabolism (17.7%), membrane transport (10.8%) and amino acid metabolism (10.4%). A higher proportion of the accessory and unique genes were related to membrane transport: 17.5% and 25%, respectively.

The orthologous gene clusters were compared with KP_SDL79 and its closely related genomes (GCA_002848605 and GCA_003031345). A total of 5082 clusters were predicted and were further divided into 4830 single-copy and 252 multi-copy protein clusters. Of these 5082 clusters, 177 were shared by at least two strains, while 44 were specific to a single strain, as shown in Figure S3.

### 3.5. Distribution of Antibiotic Resistance and Virulence Genes

**Figure 3:**
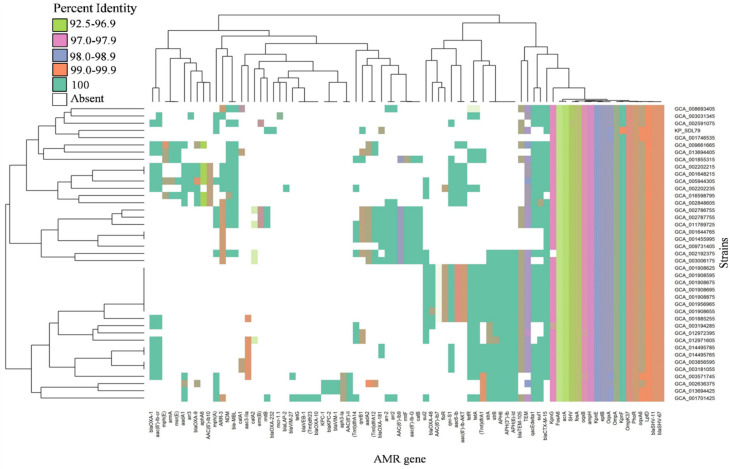
Distributions of antimicrobial determinants. The heatmaps depict the collection of AMR genes identified using CARD in 41 strains. The color density represents the varied percentage identity of each gene. The strains (*y*-axis) are hierarchically clustered using the “complete” approach with Euclidean distance based on their content of AMR genes (*x*-axis). The heatmap was generated using the R package gplots (https://github.com/talgalili/gplots, accessed on 25 January 2021).

The presence of AMR genes and encoded virulence factors were identified in this study. A total of 72 AMR genes were identified and classified into 10 classes of antibiotics based on the Antibiotic Resistance Ontology (ARO) classification system (Table S1). These were classified into major classes of antibiotics, including aminoglycosides, fosfomycin, carbapenems, macrolide, tetracycline, rifamycin, cephalosporin, peptide, quinolone and β-lactams with >90% coverage and >95% identities (Figure 3). The most prominent type of AMR determinants in all the isolates was associated with antibiotic inactivation (*ampH*, *bla*_SHV-67_, *bla*_SHV-11_, *fosA*6*, phoR*), antibiotic efflux (*lptD*, *kpnEF*, *kpnG*, *oqxAB*, *acrA*), antibiotic target alteration (*eptB*) and reduced permeability (*ompK37* and *ompA*). The presence of *bla*_OXA-232_, responsible for antibiotic inactivation in the carbapenems class, was found exclusively in KP_SDL79. The major gene family was observed in all genomes for antibiotic inactivation (59.7%) and the efflux (16.6%) mechanism.

The analysis revealed that 76 different types of virulence genes were present across all the genomes studied. The most common virulence factors included those associated with the AcrAB efflux pump, type II secretion (*T6SS*) system, type I secretion system, siderophore enterobactin (Ent), yersiniabactin, type I fimbriae, salmochelin, type II fimbriae, CPS formation and regulation. On the other hand, aerobactin was found to be the least frequent of all the virulence factors (Table S2).

### 3.6. Plasmid Prediction and Synteny Analysis

**Figure 4:**
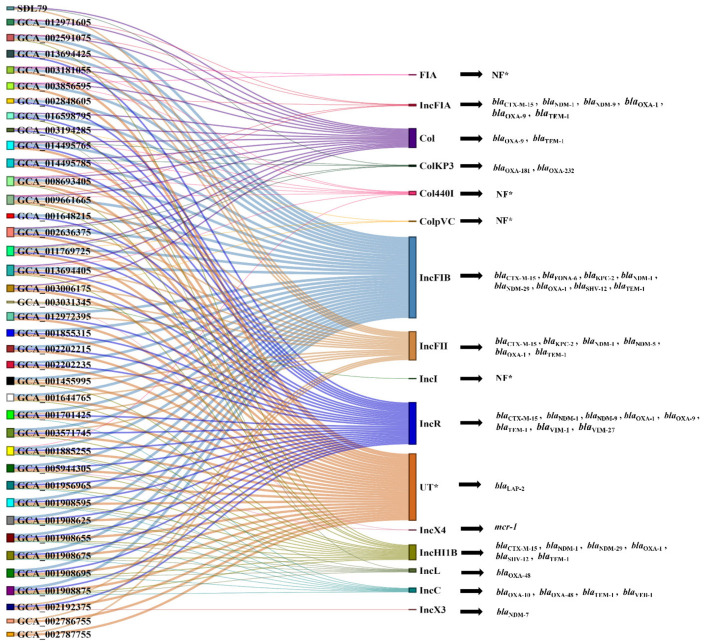
Sankey diagram depicting the distribution of the various plasmid types and their linkage to the different strains. Plasmid types found to have various AMR genes are shown and related to the strains in which the plasmid type was found (middle). Specific resistance genes are displayed on the far right that are associated with particular plasmids. The color and size of the nodes on the right is proportional to the frequency of the plasmids in ST147, respectively. Where NF* denotes not found and UT* represents untypeable plasmid.

The study found that 16 plasmid types were linked to the transfer of new phenotypic characters through horizontal gene transfer (HGT). The plasmid types were identified by linking AMR genes to different plasmid types. Col, IncFIB, IncFII, IncHIIB, IncL and IncR were often associated with the spread of extended-spectrum β-lactamases (ESBLs) (*bla*_TEM-1_, *bla*_CTX-M-15_, *bla*_SHV-12_) and carbapenemases (*bla*_OXA-48_, *bla*_OXA-10_, *bla*_VIM-27_, *bla*_NDM-1_, *bla*_NDM-29_, *bla*_LAP-2_ and *bla*_NDM-7_) (Figure 4) [39]. The *bla*_CTX-M-15_ resistance gene on the Inc plasmid type was found to be the most frequent, present in 30 out of 41 isolates, followed by *bla*_TEM-1_ (33/41), *bla*_OXA-1_ (14/41), *bla*_NDM-1_ (9/41) and *bla*_OXA-48_ (8/41).

**Figure 5:**
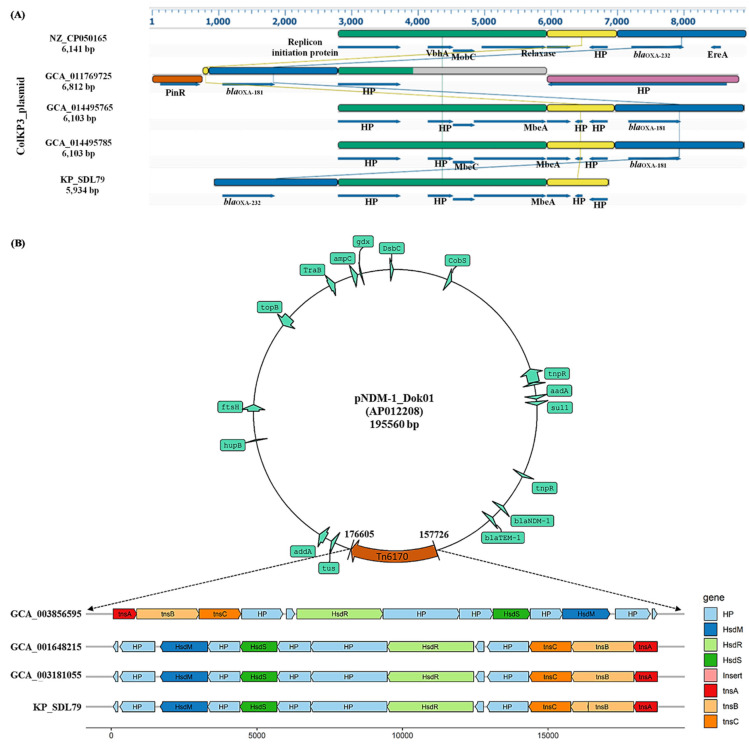
Comparative schematic representation of the ColKP3 plasmid and Tn6170 transposon element. (**A**) Genome alignments show synteny blocks obtained using Mauve alignments and visualized in DNASTAR. KP_SDL79 was compared with the strains harboring ColKP3 plasmid along with a reference plasmid sequence (NZ_CP050165). Each genome is organized horizontally, with homologous segments denoted by colored rectangles. Each identical color block denotes a genome-wide locally collinear block (LCB) or homologous region. In terms of collinearity, genomic areas were reorganized between the two genomes (KP_SDL79 and GCA_011769725). (**B**) Schematic representation of Tn*6170* compared among four isolates. Different colors are assigned to the genes, mobile elements and other features encoded based on their functional annotation. The black color “bar” at the flanking region of *tnsB* denotes a ~9 bp insert in KP_SDL79. Region of homology range is >95%. HP denotes a hypothetical protein.

Interestingly, KP_SDL79 contained plasmid types such as Col, ColKP3 and IncFII, with the unique *bla*_OXA-232_ gene found on a ColKP3 plasmid (~5934 bp). The synteny analysis of the ColKp3 plasmid showed significant shared synteny in gene arrangements with other strains (GCA_014495725 and GCA_014495785) and evidence of genetic plasticity in KP_SDL79 and GCA_011769725. A reference genome (accession number CP050165) was chosen to compare these plasmid types. The homologous regions of the plasmid were identified by aligning and linking them with linear collinear block (LCB) liners, revealing the homologous region (Figure 5A).

### 3.7. Identification of Insertion Elements and Characterization of Tn6170

The presence of forty-six insertion (IS) elements among the strains was analyzed. Out of these, twenty elements were widely distributed among all the strains with varying percentages from 10 to 100% (Figure S4). The study found that KP_SDL79 only harbors five transposon elements, with varying prevalences of ISKpn*1* (100%), IS26 (90.2%), IS*6100* (49%), ISKpn*14* (26.8%) and Tn*6170* (10%) among the 41 strains. The least explored mobile genetic element, Tn*6170*, was only present in KP_SDL79, a putative 18.8-kilobase-long transposon of the Tn*7*-like family. This was first reported in *E. coli* in 2014 and is carried by a 195,560 bp plasmid pNDM-1_Dok01 (Accession number: AP012208.1) [40]. This element contains the *hsdR* operon and three heteromeric transposase genes, *TnsABC,* responsible for site-specific transposition [41]. The synteny analysis showed similar gene arrangements in the genomes with the Tn*6170* element, except for KP_SDL79 in which a 9 bp insert in the *tnsB* gene was exclusively found (Figure 5B). This suggests the possibility of a loss or gain in function during the transposition process, which requires further investigation and validation in vitro.

### 3.8. Prediction of Serotypes, Prophages and CRISPR-Types

Among the isolates, five different KL types were found, including KL64 (85.3%), KL51 (2.5%), KL20 (2.5%), KL122 (2.5%) and KL10 (7.3%) (Figure 2). In addition, three O types were predicted among the isolates, including O3/O3a (4.3%), O2v2 (4.8%) and O2v2 (87.8%). A common set of lysogenic phages were present in almost all strains (38/41, with the exception of three isolates: GCA_002591075, GCA_003194285 and GCA_009661665) and carried a 34 Kbp intact phage sequence, ST147-VIM1phi7, belonging to the *Myoviridae* family. The CRISPR/Cas arrays were found in 28 genomes, classified as subtype I–E with an average direct repeat length of 37.5 bp and containing eight types of Cas proteins, including Cas1, Cas2, Cas3, Cas5, Cas6, Cas7, Cse1 and Cse2 (Table S3).

### 3.9. ST147 Isolates Have Significant and Diverse Biosynthetic Potential

A total of six different types of biosynthetic gene clusters (BGCs) were found, including those encoding for NRPS (40), NRPS-like (1), redox-cofactor (38), TIPKS (21), Ripp-like (27) and thiopeptide (39), which accounted for 66% of the total genomes (Figure S5). The strain GCA_002848605 had a unique NRPS-like cluster. Virulence factors such as *entA* and *entS* were found in the NRPS, thiopeptide and Ripp-like gene clusters, while *Irp1* and *Irp2* were found in the TIPKS cluster. Major facilitator superfamily (MFS) efflux pump proteins were co-localized in the redox-cofactor and thiopeptide clusters. The BGCs contained a large collection of core genes (3253), categorized as Enterobactin esterase, Catalase-peroxidase, Enterobactin transporter (*entS*), transcriptional regulatory protein (*ompR*), type II toxin-antitoxin, *YcaO*-like family, MFS and ABC transporter types. Putative cytoplasmic transmembrane protein (*YihE*), type IV secretion system protein (*virB1*), p-type conjugative transfer protein (*virB9*) and iron-regulatory protein (*Irp1*) were classified among the 819 accessory genes (Table S4).

### 3.10. Core SNP Identification and Phylogenetic Reconstruction

A total of 5502 recombination-free core SNPs with one variation occurring every 965 bases and 34 multi-allelic mutations were detected based on the alignment to the reference genome (GCA_016903735). Of these SNPs, 1459 were non-synonymous and 3235 were synonymous types of substitutions. Approximately 45% of the SNPs were located in the upstream and downstream coding sequence (CDS) regions, while 8% were in the coding region. The dN/dS ratio of nonsynonymous substitutions per nonsynonymous site (pN) to the number of synonymous substitutions per synonymous site (pS) was 0.45, indicating negative Darwinian selection. Trends of positive Darwinian selection produce dN/dS > 1, whereas tendencies of negative Darwinian selection, or the selective removal of detrimental alleles, produce dN/dS < 1 [42]. There were 8723 transition and 4524 transversion mutations with a ratio of 1.9. A phylogenetic analysis was conducted using consensus core genome sequences, which revealed the grouping of strains into three sub-lineages based on shared core SNPs. The branch lengths are shown with the SNP variations and node labels indicating overall SNPs among the strains (Figure S6). Two strains from Thailand and Switzerland (GCA_003031345 and GCA_002848605) showed a close relationship to the strain KP_SDL79 regarding the highest number of shared SNPs (1403) within the inner nodes of the clusters, highlighting the diversity of the strains across geographical regions.

### 3.11. Bayesian Phylogenetic Analysis

**Figure 6:**
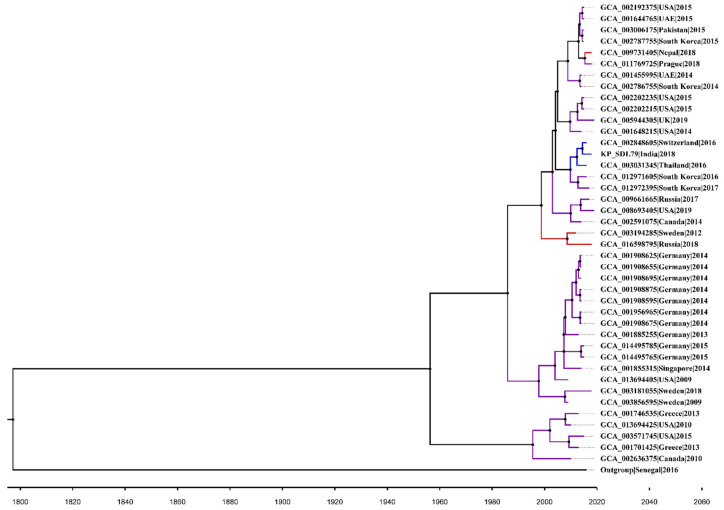
Bayesian inferred phylogenetic assignment of the ST147 isolates. The phylogeny with Bayesian dates is presented. According to their evolutionary relationships, the 41 genomes were divided into three clusters obtained from fastbaps. Different colors are used to represent the clusters. The tips label of the tree includes strain accession/name, country and isolation year. The time tree was created using FigTree.

The divergence rate was estimated for the ST147 isolates using a Bayesian timescale analysis. The root-to-tip regression analysis indicated limited clock-like behavior (R^2^ = 0.23, *p*-value < 0.005) in the ST147 population, with only 23% of the variation described by time. This could be due to a narrow range of sampling (2009–2018) and sample size (n = 42, including an outgroup of *Klebsiella africana,* strain 200023, accession: GCA_020526085). The present analysis suggests that a molecular clock analysis is appropriate for the core genome-based rate estimation due to regression’s positive slope and clock-like signal detection. The GTR model was found to be the best-fitting nucleotide substitution model. A BEAST analysis of the core genome sequence was performed using both strict and relaxed molecular clock models. After running an altered combination of demographic and molecular clock models, a Bayesian Skyline demographic model and uncorrelated lognormal relaxed clock were finally chosen as the best-fitting model (Table S5). The substitution rate for the ST147 population was estimated at 5.9 × 10^−3^ per site per year (95% HPD = 1.1082 × 10^−4^ to 0.0126). The initial divergence of the ST147 isolates was in 1951 and the most recent common ancestor (TMRCA) for the entire *Kp* population was estimated to be in 1621 (Figure 6).

## 4. Discussion

The outcomes of the current study provided valuable insights into the genetic variation and evolution pattern of *KP* ST147, a fast-spreading HiR clone [5]. Despite the significance of this clone, there has been limited research on its worldwide spread over time. The discovery of the extensively drug-resistant (XDR) *Kp* phenotype belonging to ST147 in India encouraged us to perform a detailed examination of the intraspecific genomic features and evolutionary dynamics of ST147. The present study encompassed the phylogenetic diversity and genomic adaptability of ST147 strains, including the acquisition of important determinants, such as virulence factors, antibiotic resistance genes, plasmids, transposons and prophages. Herein, a pangenome analysis was performed using only complete genome sequences from a public database from geographically dispersed regions, as incomplete assemblies could result in incorrect annotations and inaccurate estimations of gene evolution rates, which have already been attempted [43]. The ST147 genomes have a diverse structure, with 45.8% of the genes shared across all strains (core genes) and 54.1% being unique to each strain (accessory genes), which reveals a high level of genome plasticity (Figure S1). This resembled the pangenome structure of *Klebsiella aerogenes* [44].

Similarly, the pangenomes of ST11 *Kp* showed an open structure, indicating a higher degree of genetic diversity [45]. A maximum-likelihood (ML) phylogenetic tree was built using recombination-free core genome sequences. Forty-one ST147 *KP* genomes were clustered into three major groups, consistent with the previous report [46]. The strain KP_SDL79 clustered separately along with the strains isolated from wastewater (Switzerland, GCA_002848605) and rectal swabs (Thailand, GCA_003031345) [47], emphasizing its habitat diversity (Figure 2). Prior research showed that the ST147 clone was widespread in countries such as Greece, China, Slovenia, and Singapore, and its spread could be attributed to anthropogenic activities or contamination from biomedical waste [5].

According to a previous report, the highest number of AMR genes were detected in strains belonging to ST147 [48]. This study revealed the presence of 72 prominent types of AMR genes belonging to almost all classes of antibiotics, signifying a global health concern. These findings were supported by the results of Sundaresan et al., 2022 who found that the inactivation of antibiotics and efflux mechanisms had the highest prevalence among the strains [48]. *Kp* is known to cause virulence in humans and is associated with high mortality rates in immunocompromised patients [49]. It is classified into two strains: the classical strain (cKp) and the hypervirulent strain (hvKp). The hvKp strain is differentiated from the cKp strain by the presence of *rmpA* and *rmpA2* mucoid-regulator genes, K1, K2, and K20 capsular types, and aerobactin [50]. In this study, most of the virulence factors in the isolates were associated with type I/II secretion systems, ent siderophores, yersiniabactin, type I/II-fimbriae, salmochelin, CPS formation and efflux pump (Table S2), indicating high pathogenicity potential [51]. These strains were classified as cKp strains, as they lack the genetic characteristics of hvKp strains. The study also found 16 types of plasmids, with IncFIB, IncR, IncFII and Col being the most widespread and carrying mostly β-lactamase genes. According to previous findings, the spread of *bla*_CTX-M-15_ and *bla*_SHV-12_ in *Kp* is largely facilitated by IncR plasmids only [52]. The *bla*_OXA-181_ gene was found on a ColKp3 plasmid in ST147 strains from the Czech Republic and Germany, but KP_SDL79 was the only strain with the *bla*_OXA-232_ gene on a ColKp3 plasmid. This differs from a recent report from India, which showed the presence of *bla*_OXA-232_ on ColKp3 plasmids in ST231 *Kp* clones [53]. To our knowledge, this is the first report of the *bla*_OXA-232_ gene being present on a non-conjugative plasmid in the ST147 strain. Comparing the *bla*_OXA_ genes on the ColKp3 plasmid among closely related KP_SDL79 strains revealed a substantial difference in their genetic arrangement, suggesting the possibility of recombination events. A recent study proved that significant recombination events have occurred in the ST147 and ST37 lineages, indicating a crucial role in the emergence of epidemiologically significant clones [54].

The capsular serotypes in *Kp* are the prominent phylogenetic markers. Most of the isolates were found to have the KL64 capsular serotype (85.3%), while some belonged to the KL10 (7.3%) type. According to a previous report, the Tuscan outbreak clone belonged to a different sub-clade of ST147 and was found to carry the KL64 capsular locus, which could play a pivotal role in determining a virulent phenotype [55]. Multiple clonal expansions of sub-lineages with different KL or O-types impact diagnostic and therapeutic measures, such as immunization and phage therapy [56]. Prophages are critical to bacterial evolution as they carry the genetic material acquired through horizontal gene transfer events [57]. In these findings, 92.6% of *Kp* isolates were found to have complete *Myoviridae* family prophages that affect the host’s resistance and virulence properties. A previous report also identified these prophages in 90% of ST147 genomes [7].

The discovery of the T*n6170* transposon element in *Kp* is noteworthy, as it was identified for the first time with an insertion in the flanking region of *tnsB* integrated into the chromosome. This *Tn7*-like family transposon was previously reported in *E. coli*, carried by plasmid pNDM-1_Dok01 [58]. However, its transfer mechanism to *Klebsiella* remains unknown and requires further investigation.

The study of BGCs in ST147 strains revealed the presence of various virulence factors and significant intra-species variations. Several BGCs were frequently observed, responsible for producing NRPS, redox-cofactor, TIPKS, Ripp-like and thiopeptide. Most BGCs were linked to virulence factors, such as the Enterobactin transporter and iron-regulatory protein. These findings are consistent with previous research on *Steptomyces* sp. by Belknap et al., 2020 [59]. According to a previous study, the most prevalent BGCs in *Klebsiella* sp. were those responsible for producing bacteriocins and associated with a virulence factor. However, the bacteriocin gene cluster appeared to be incomplete [21]. These findings suggest that the prediction of secondary metabolites may provide a potential target for therapeutic development, presenting a promising opportunity to discover a new drug. *Kp* can interact with other microorganisms in the microbiota, leading to potential changes in its bioactivity. Studies have shown that this organism can form mixed-species biofilms with other relevant organisms, such as *Pseudomonas aeruginosa,* and can often co-exist in the environment. Additionally, other microbiota members can influence *Kp*‘s virulence and pathogenesis [60]. It is crucial to understand these interactions to formulate approaches that can manage *K. pneumoniae* infections and facilitate healthy microbiota.

The evolution of the bacterial genome has been significantly influenced by CRISPR-associated proteins, which are also crucial for the diversification of the genome [61]. The subtype I–E is consistent in all isolates, which aligns with the findings of Zemmour et al., 2021 [62]. The analysis of divergent SNPs across the “complete” genomes allowed for a deeper understanding of the genetic basis of phenotype variability [63]. In the present study, the negative Darwinian selection rate in the core genes indicated that the mutations are not yet stabilized in the population and the process of purifying the genome through the removal of harmful alleles is ongoing, resulting in the adaptation and evolution of clones [64]. In this study, complete genome sequences were utilized to evaluate the evolutionary history of ST147, resulting in a better phylogenetic and temporal resolution [65]. Intriguingly, the results showed a faster evolutionary rate of 5.9 × 10^−3^ substitutions/site/year, which was in contrast to the previously reported slower rate of 1.03 × 10^−6^ substitutions/site/year for global lineages of ST147 [7]. The observed discrepancy may be attributed to the use of draft and scaffold-level genome assemblies in the previous studies. The utilization of complete genomes in the BEAST analysis improved the accuracy and precision of the evolutionary history estimation. The evolutionary timeline study of ST147 strains showed that they diverged earlier than the isolation date, potentially due to transmission from the environment to clinics or vice versa. However, accumulating a bigger dataset is needed to confirm this hypothesis.

## 5. Conclusions

In conclusion, the results of this study provide insights into the genetic diversity and evolutionary dynamics of the HiR clone of *Kp*, a major global health concern due to its high prevalence and rapid emergence of antibiotic resistance. The pangenome analysis revealed genome plasticity and openness, with a large number of accessory genes. The presence of numerous antibiotic resistance and virulence factors highlights the pathogenicity of this clone. The detection of transposon elements further establishes their transmission ability. Repeated outbreaks of these HiR clones might pose a threat to society. Hence, molecular surveillance is the need of the hour to prevent an uncontrollable epidemic.

Further studies on inter-clonal diversity will be essential to gain a more comprehensive understanding of this clone’s outbreak and develop effective therapeutic interventions. Continued efforts in the complete genome sequencing of ST147 are also necessary for evaluating its pattern of evolution and host–plasmid interactions, ultimately paving the way from a genome to a drug approach for tackling *Kp* infections. Overall, this study underscores the urgent need for molecular surveillance to combat the spread of antibiotic resistance and prevent the emergence of these HiR clones.

## Figures and Tables

**Table 1:** The summary of country, host, isolation source, collection year and basic genome statistics of all the strains used in this study.

Accession	Country	Host	Isolation Source	Collection Year	Chromosome	Genome Size (bp)	GC Content (%)	CDS	rRNA	tRNA	misc_RNA	tmRNA	K-Locus	O-Locus
GCA_001455995	United Arab Emirates	Human	Urine	2014	7	5980203	57.33	5631	25	89	130	1	KL64	O2v1
GCA_001644765	United Arab Emirates	Human	Urine sample	2015	6	6088457	57.29	5736	25	89	132	1	KL64	O2v1
GCA_001648215	USA	Human	Perirectal swab	2014	4	5566588	57.43	5101	25	87	112	1	KL64	O2v1
GCA_001701425	Greece	Human	Stool	2013	6	5760711	57.58	5403	25	88	122	1	KL64	O2v1
GCA_001746535	Greece	Human	Rectal swab	2013	1	5344576	57.42	4946	31	103	116	1	KL64	O2v1
GCA_001855315	Singapore	Human	Hospital	2014	5	5718854	57.3	5297	25	90	110	1	KL64	O2v1
GCA_001885255	Germany	Human	Nose	2013	7	5920942	57.36	5559	25	88	120	1	KL64	O2v1
GCA_001908595	Germany	Human	Tracheal secretion	2014	6	5916317	57.36	5550	25	88	119	1	KL64	O2v1
GCA_001908625	Germany	Human	Abdominal drainage fluid	2014	6	5907691	57.36	5541	25	88	118	1	KL64	O2v1
GCA_001908655	Germany	Human	Nasal swab	2014	6	5913496	57.36	5551	25	88	119	1	KL64	O2v1
GCA_001908675	Germany	Human	Abdominal drainage fluid	2014	6	5921376	57.36	5553	25	88	119	1	KL64	O2v1
GCA_001908695	Germany	Human	Anal swab	2014	6	5914083	57.36	5543	25	88	119	1	KL64	O2v1
GCA_001908875	Germany	Human	Abdominal drain fluid	2014	6	5920288	57.36	5551	25	88	119	1	KL64	O2v1
GCA_001956965	Germany	Human	Anal swab	2014	5	5869161	57.36	5497	25	88	118	1	KL64	O2v1
GCA_002192375	USA	Human	Medical	2015	5	5708112	57.3	5320	25	89	120	1	KL64	O2v1
GCA_002202215	USA	Human	Medical	2015	5	5656270	57.41	5238	25	88	116	1	KL64	O2v1
GCA_002202235	USA	Human	Medical	2015	6	5782129	57.42	5383	25	88	120	1	KL64	O2v1
GCA_002591075	Canada	Human	Urine	2014	6	5752865	57.56	5369	25	86	121	1	KL64	O2v1
GCA_002636375	Canada	Human	Blood	2010	7	5727717	57.45	5397	25	90	118	1	KL64	O2v1
GCA_002786755	South Korea	Human	Bronchial washing	2014	4	5720338	57.32	5346	25	89	129	1	KL64	O2v1
GCA_002787755	South Korea	Human	Peritoneal fluid	2015	4	5707921	57.32	5317	25	89	128	1	KL64	O2v1
GCA_002848605	Switzerland	Environment	Waste water	2016	3	5630984	57.4	5428	25	87	122	1	KL10	O3/O3a
GCA_003006175	Pakistan	Human	Trachaeal secretion	2015	8	5752275	57.29	5368	25	89	128	1	KL64	O2v1
GCA_003031345	Thailand	Human	Rectal swab	2016	4	5640638	57.42	5292	25	86	123	1	KL10	O3/O3a
GCA_003181055	Sweden	Human	Unknown	2018	5	5657209	57.27	5220	25	88	116	1	KL64	O2v1
GCA_003194285	Sweden	Human	Urine	2012	5	5391141	57.54	5005	28	87	117	1	KL122	O2v2
GCA_003571745	USA	Human	Medical	2015	6	5768716	57.54	5375	25	89	124	1	KL64	O2v1
GCA_003856595	Sweden	Human	Faeces	2009	5	5657200	57.27	5219	25	88	116	1	KL64	O2v1
GCA_005944305	United Kingdom	Human	Rectal swab	2019	8	6109775	57.34	5828	25	88	119	1	KL64	O2v1
GCA_008693405	USA	Human	Clinical isolate	2019	8	5642810	57.48	5249	24	87	120	1	KL64	O2v1
GCA_009661665	Russia	Human	Urine	2017	6	5645610	57.46	5244	25	88	115	1	KL64	O2v1
GCA_009731405	Nepal	Human	Human sputum	2018	1	5422388	57.28	5006	25	88	110	1	KL51	O2v2
GCA_011769725	Czech Republic	Human	Rectal swab	2018	11	5919785	57.33	5514	25	90	136	1	KL64	O2v1
GCA_012971605	South Korea	Human	Blood	2016	6	5820688	57.35	5432	25	88	123	1	KL64	O2v1
GCA_012972395	South Korea	Human	Blood	2017	9	5688247	57.36	5308	25	86	113	1	KL64	O2v1
GCA_013694405	USA	Human	Clinical isolate	2009	8	5793006	57.46	5412	25	86	123	1	KL64	O2v1
GCA_013694425	USA	Human	Clinical isolate	2010	5	5610747	57.44	5237	25	88	115	1	KL64	O2v1
GCA_014495765	Germany	Human	Rectal swab	2015	9	5730837	57.24	5315	25	89	120	1	KL64	O2v1
GCA_014495785	Germany	Human	Throat	2015	9	5729180	57.24	5303	25	89	120	1	KL64	O2v1
GCA_016598795	Russia	Human	Urine	2018	4	5797747	57.41	5434	25	88	117	1	KL20	O2v1
GCA_024124405	India	Human	Urine	2018	43 *	5622734	57.21	5266	3	84	112	1	KL10	O3/O3a

* Contigs.

**Table 2:** Quality assessment of the de novo assemblies generated from 4 different assemblers for the KP_SDL79 strain. All statistics are based on contigs with a size ≥ 200 bp. All draft assemblies pass the completeness test with a score of 99.19.

Features	*K. pneumoniae* HS11286	Unicycler	AbySS	SOAPdenovo	SPAdes
Contigs (≥1000 bp)	1	65	119	129	132
Contigs (≥50,000 bp)	1	23	40	41	24
Largest contig size (bp)	5,333,942	714,080	323,583	390,642	1,005,571
Total length (bp)	5,333,942	5,562,754	5,833,980	5,815,984	5,988,665
GC Content (%)	57.48	57.21	56.82	56.78	56.52
N50 (bp)	5,333,942	218,233	113,012	105,915	218,806
N75 (bp)	5,333,942	147,654	66,133	61,400	129,870
L50 (bp)	1	7	17	18	8
L75 (bp)	1	15	33	35	17
Genome fraction (%)	100	91.557	91.018	91.133	91.022
Ns per 100 kbp	0	325.46	1462.79	1892.94	1879.7
Mismatches per 100 kbp	0	675.84	681.03	679.32	677.46
Complete Gene	5404	4736	4702	4726	4727
Partial Gene	0	84	87	94	95
Contamination	0	1.46	2.23	2.22	2.22

## Data Availability

The raw sequencing reads of KP_SDL79 are available in the National Center for Biotechnology Information (NCBI) under the accession number JAMXTJ000000000. The other analyzed genomes are available in the GenBank database (https://www.ncbi.nlm.nih.gov/genbank/, accessed on 25 January 2021).

## Supplementary Materials

The following supporting information can be downloaded at: https://www.mdpi.com/article/10.3390/genes14051037/s1, Figure S1: Statistics of ST147 pangenome; Figure S2: Functional profiling of pan-genes; Figure S3: Venn diagram showing the gene-content similarity in terms of common, shared and unique of KP_SDL79 with its most closely related genome; Figure S4: Heatmap showing transposons elements presence (red) and their absence (blue); Figure S5: Distribution of BGCs were identified in all strains using antiSMASH; Figure S6: A phylogenetic tree demonstrating the genetic link between the genomes based on core SNPs; Table S1: The identified AMR determinants in all 41 strains along with gene family, drug class, resistantant mechanism and percentage identity; Table S2: Details of virulence factros and its presence (1)/abscence (0) found in 41 analyzed genomes; Table S3: CRISPR/Cas types and distribution among all the strains; Table S4: Summary of biosynthtic gene clustres found in all the analyzed strains; Table S5: A summary of the evolutionary clock rate obtained for each of the models in BEAST analysis.
