# Supplementary material for: Unravelling the Evolutionary Dynamics of High-Risk Klebsiella pneumoniae ST147 Clones: Insights from Comparative Pangenome Analysis
Source: Genes (Basel). 2023 May 2;14(5):1037. doi: 10.3390/genes14051037 (PMC10218137; doi:10.3390/genes14051037)
Supplement: Supplementary file 1 [file genes-14-01037-s001.zip › supplemantry_figures.pdf]

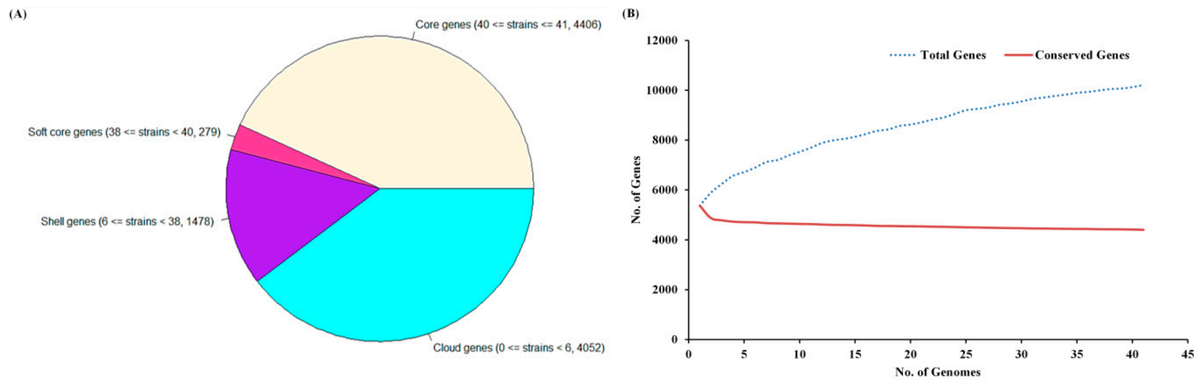

Figure S1. Statistics of ST147 pangenome. (A) The pangenome components summarised based on the presence of individual genome features. (B) The ST147 pangenome has a gene accumulation curve that shows the amount of conserved genes vs total genes in 41 genomes. Total genes represent the expansion of the pangenome as newly discovered genes were incorporated as individual genomes were added one by one. Conserved genes are an adjustment to the core genome size depending on the inclusion of each isolate's genome.

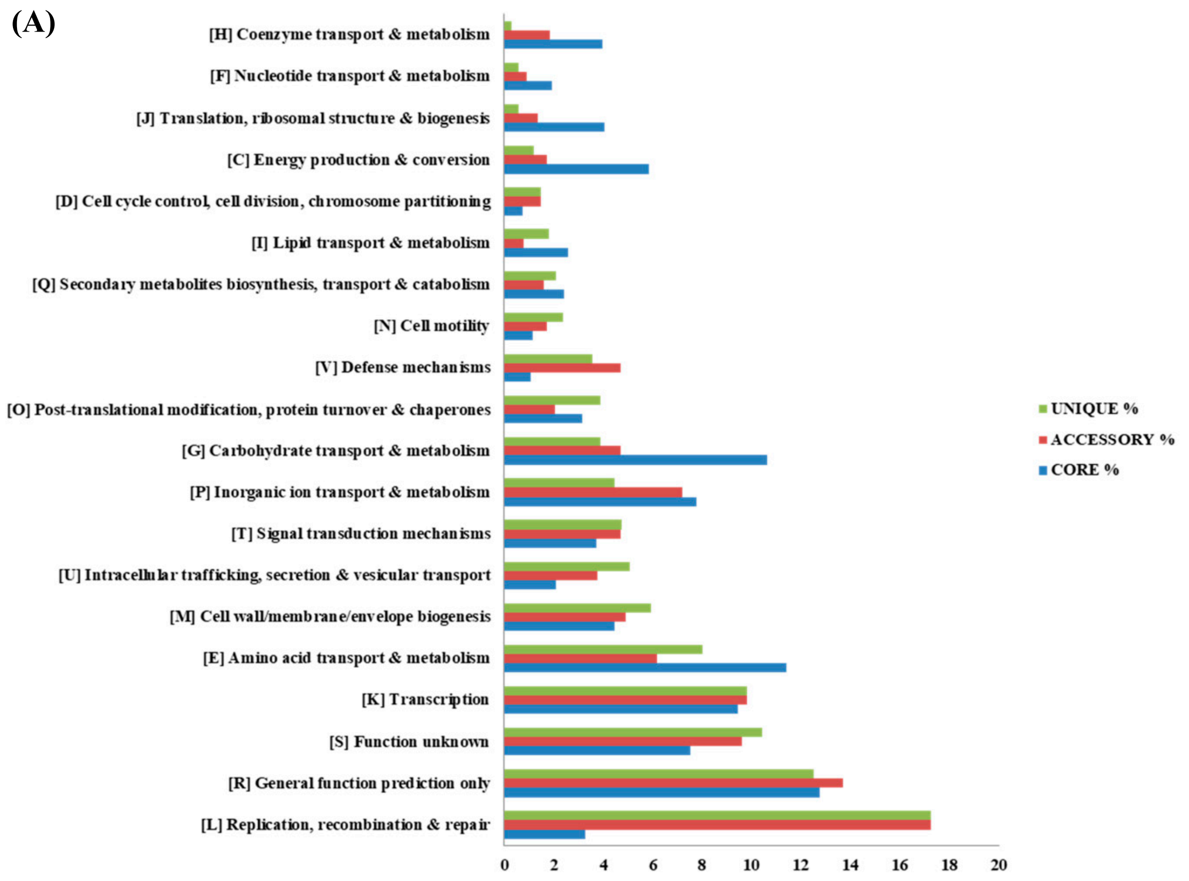

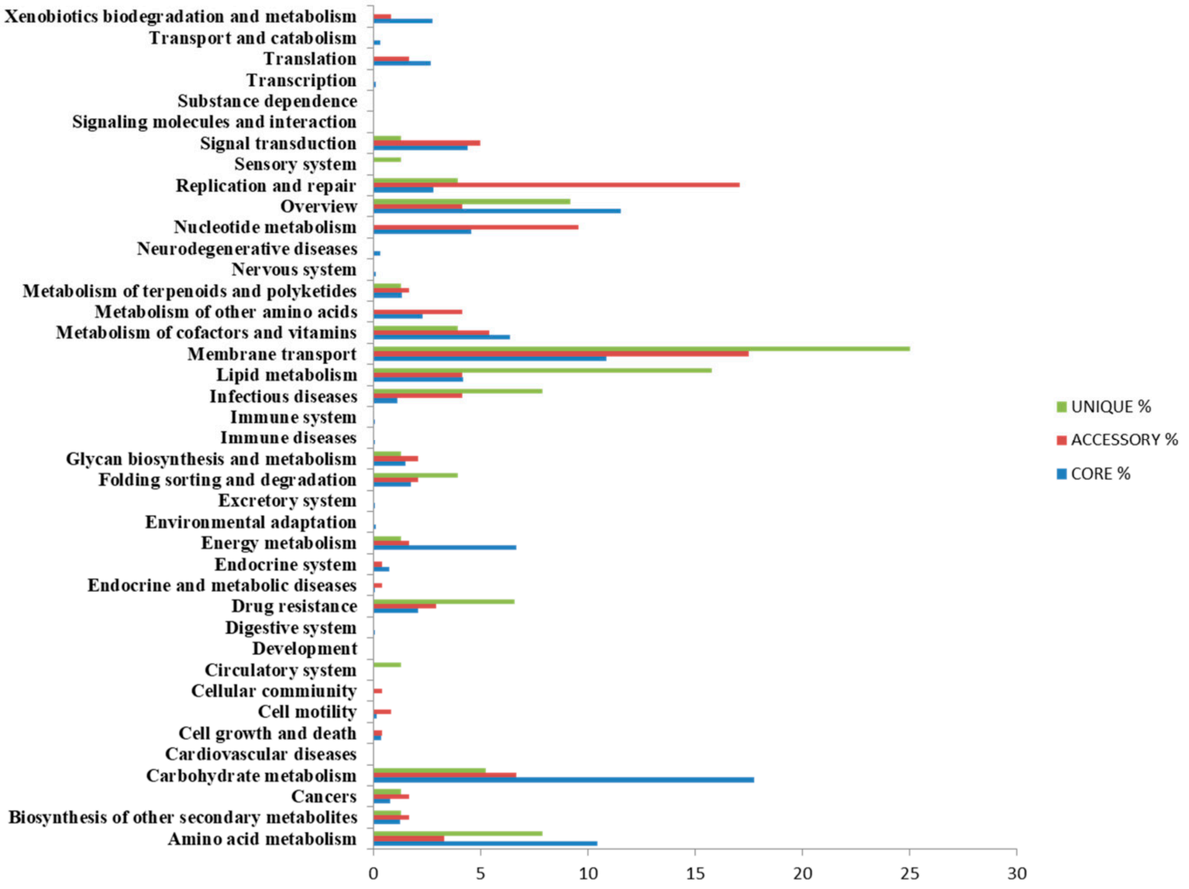

Figure S2 (A). Functional profiling of pan-genes using COG; **(B).** Functional profiling of pan-genes using KEGG.

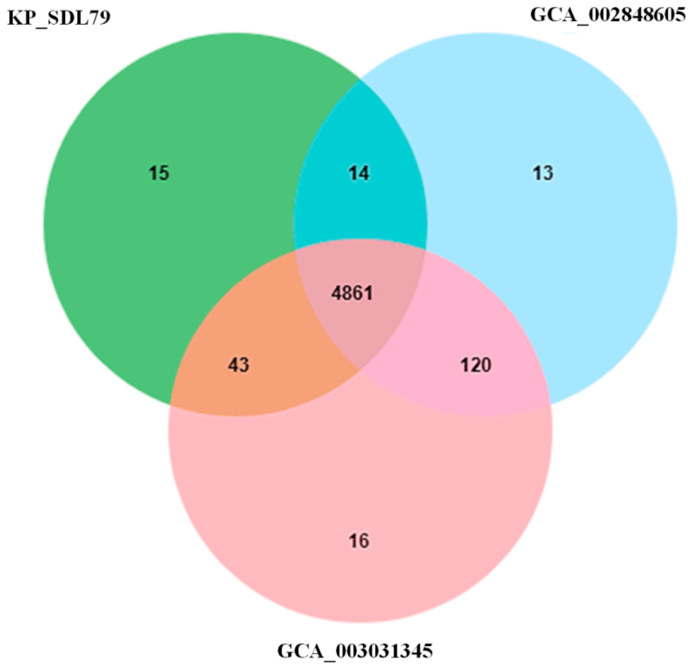

Figure S3. Venn diagram showing the gene-content similarity in terms of common, shared and unique of KP_SDL79 with its most closely related genome. The illustration is created using OrthoVenn2.

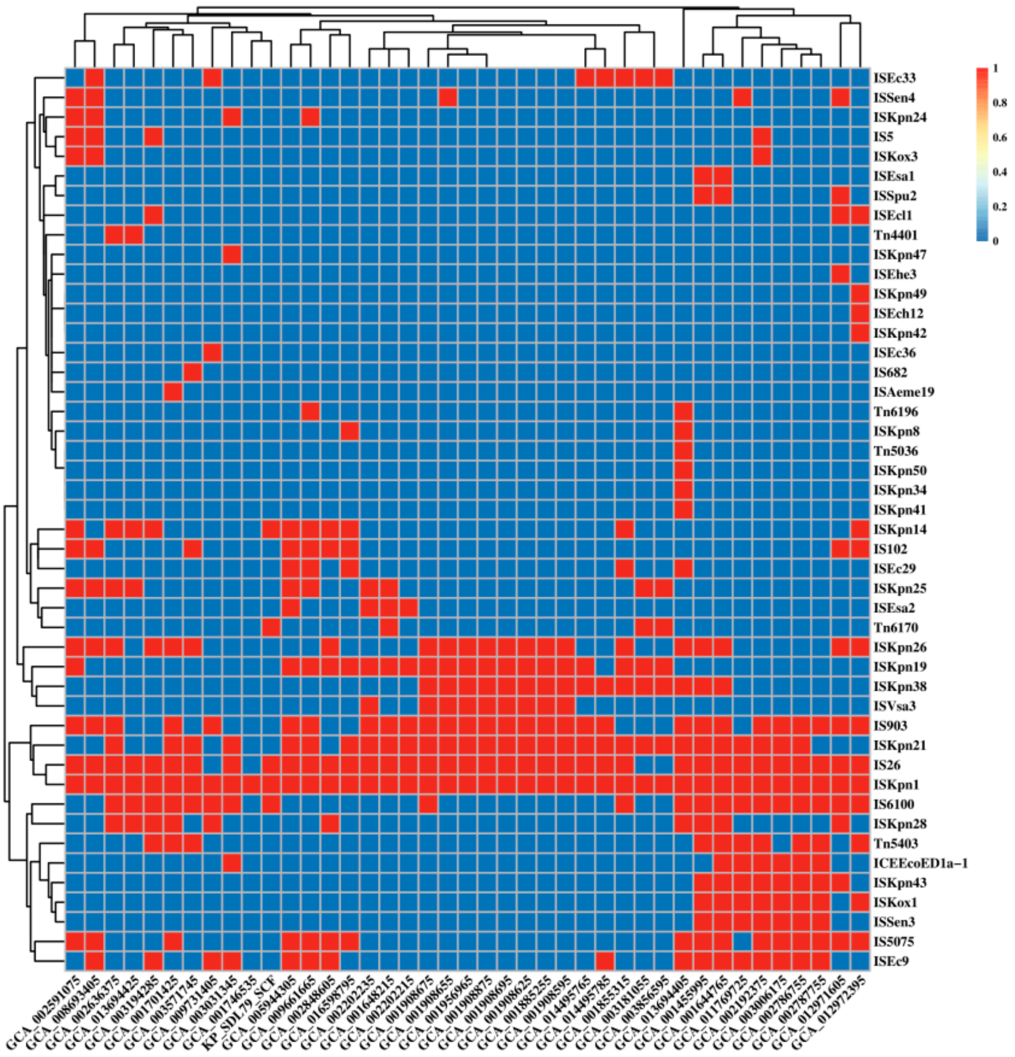

Figure S4. Heatmap showing transposons elements presence (red) and their absence (blue). The presence/absence data of the genes are used to create the cluster diagram on the left.

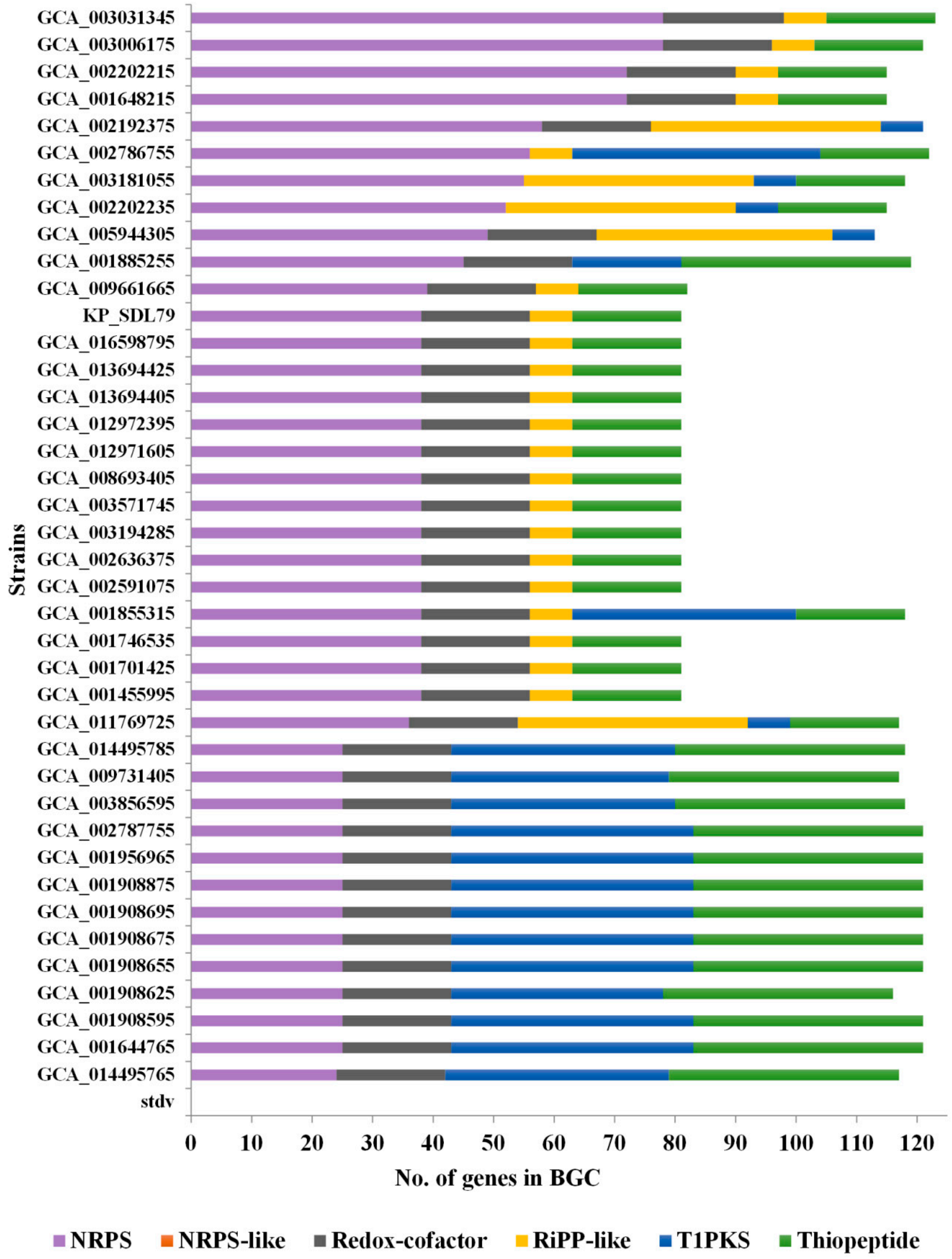

Figure S5. Distribution of BGCs were identified in all strains using antiSMASH. GCA_002848605 is the only strain containing the NRPS-like BGC.

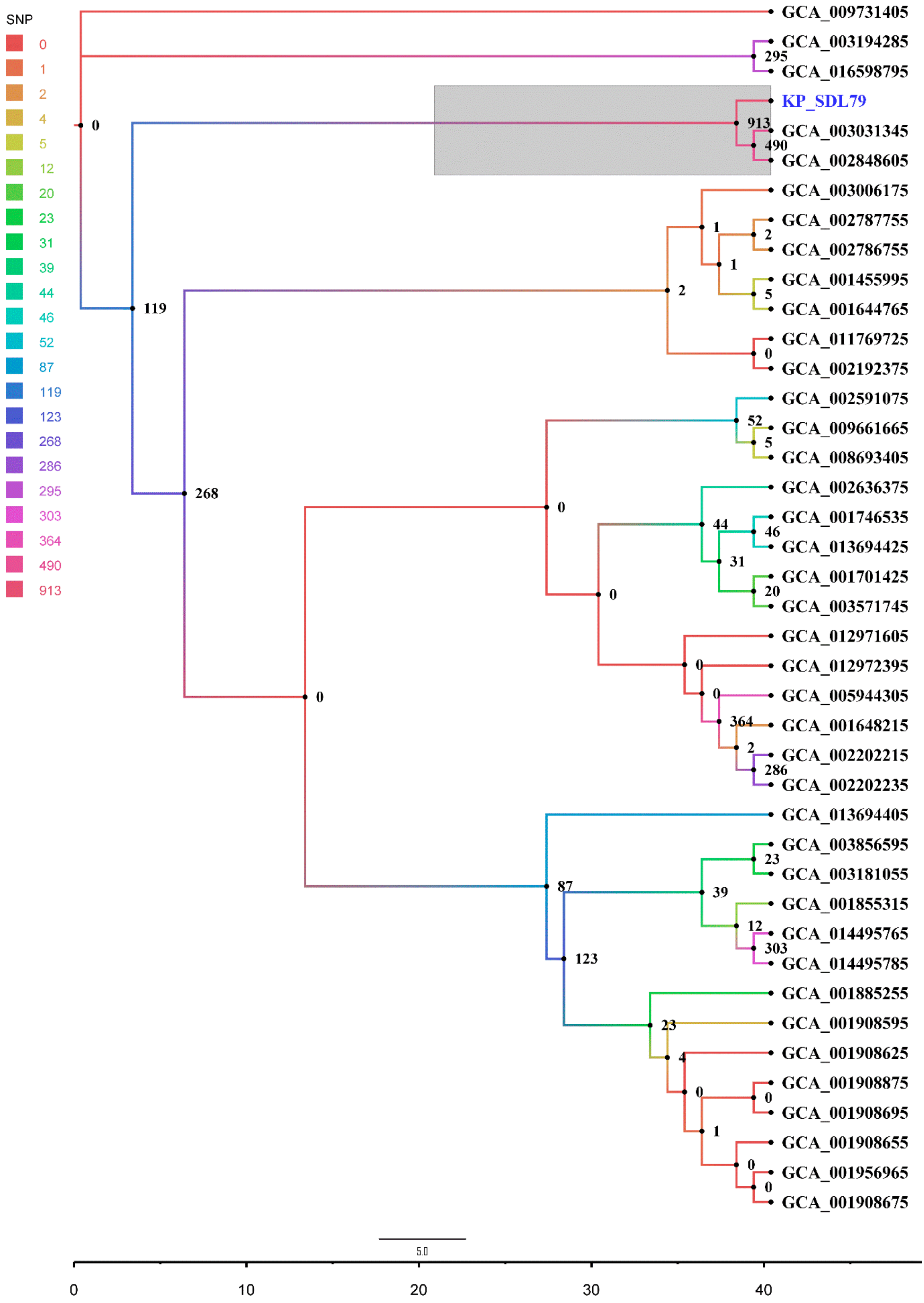

Figure S6. A phylogenetic tree demonstrating the genetic link between the genomes based on core SNPs. Based on varied SNPs the tree has split into three distinct clusters of different origins. The SNPs shared in the inner node of the clusters varied from 0 to 913. Strikingly

KP_SDL79 showing genetic relatedness with the strains (GCA_003031345 and GCA_002848605), sharing highest number of SNPs (913) in the inner node (highlighted the cluster in grey colour). Colouring of branches based on the SNPs shared in the inner node of the tree.
