# Supplementary material for: Unravelling the Evolutionary Dynamics of High-Risk Klebsiella pneumoniae ST147 Clones: Insights from Comparative Pangenome Analysis
Source: Genes (Basel). 2023 May 2;14(5):1037. doi: 10.3390/genes14051037 (PMC10218137; doi:10.3390/genes14051037)
Supplement: Supplementary file 1 [file genes-14-01037-s001.zip › Supplementary_TableS5.pdf]

Supplementary Table S5: A summary of the evolutionary clock rate obtained for each of the models in BEAST analysis.

Parameters	Strict Clock		Uncorrelated relaxed Clock					
	Skyline	Skygrid	Skyline			Skygrid		
			lognormal	gamma	exponential	lognormal	gamma	exponential
Mean	4.80 x10-5	1.53 x10^{-6}	6.58 x10^{-3}	1.21 x10^{-4}	2.56 x10^{-4}	5.93 x10^{-3}	3.30 x10^{-4}	1.89 x10^{-5}
Std. of mean	5.22 x10-5	4.14 x10^{-7}	3.49 x10^{-4}	3.37 x10^{-6}	1.11 x10^{-5}	2.43 x10^{-4}	9.66 x10^{-6}	6.40 x10^{-6}
Stdev.	4.14 x10^{-5}	6.83 x10^{-6}	4.28 x10^{-3}	2.52 x10^{-5}	1.47 x10^{-4}	3.53 x10^{-3}	6.31 x10^{-5}	8.28 x10^{-5}
Variance	1.72 x10^{-9}	4.67 x10^{-11}	1.83 x10^{-5}	6.35 x10^{-10}	2.16 x10^{-8}	1.24 x10^{-5}	3.98 x10^{-9}	6.85 x10^{-9}
Median	4.26 x10^{-5}	1.56 x10^{-11}	5.80 x10^{-3}	1.18 x10^{-4}	2.35 x10^{-4}	5.41 x10^{-3}	3.25 x10^{-4}	2.16 x10^{-11}
Value range	[2.6413 x10^{-14}, 2.4992 x10^{-4}]	[2.7432 x10^{-34}, 1.3039 x10^{-4}]	[1.643 x10^{-5}, 0.029]	[3.1686 x10^{-5}, 2.2677 x10^{-4}]	[1.0228 x10^{-7}, 1.1107 x10^{-3}]	[9.0478 x10^{-6}, 0.0295]	[1.7571 x10^{-4}, 4.8942 x10^{-4}	[2.3661 x$10^{-4\ 8}$, 1.3272 x10^{-3}]
Geometric mean	7.76 x10^{-6}	6.96 x10^{-13}	5.05 x10^{-3}	1.19 x10^{-4}	2.05 x10^{-4}	4.78 x10^{-3}	3.24 x10^{-4}	9.36 x10^{-13}
95% HPD interval	[2.6413 x10^{-14}, 1.2255 x10^{-4}]	[2.7432 x10^{-34}, 7.9661 x10^{-6}]	[2.0114 x10^{-5}, 0.0147]	[7.5745 x10^{-5}, 1.8481 x10^{-4}]	[3.2822 x10^{-7}, 5.3588 x10^{-4}]	[1.1082 x10^{-4}, 0.0126]	[2.3647 x10^{-4}, 4.5442 x10^{-4}]	[2.3661 x10^{-48}, 9.3881 x10^{-5}]
Auto-correlation time (ACT)	1.43 x10^{6}	3.31 x10^{5}	5.99 x10^{5}	1.61 x10^{6}	5.13 x10^{5}	4.28 x10^{5}	2.11 x10^{6}	5.38 x10^{5}
Effective sample size (ESS)	63	272	150.2	56.1	175.6	210.1	42.6	167.2
